# The effect of consumption inequality on subjective well-being: Evidence from China

**DOI:** 10.1371/journal.pone.0310193

**Published:** 2024-11-19

**Authors:** Tiantian Dong, Xu Ye, Zhonggen Mao

**Affiliations:** Institute of Western China Economic Research, Southwestern University of Finance and Economics, Chengdu, Sichuan, China; Xi’an Jiaotong University, CHINA

## Abstract

As an essential dimension of economic inequality, consumption inequality is tightly associated with public welfare. This study investigates the effect of consumption inequality on individuals’ subjective well-being (SWB) in China using data from the 2014, 2018, and 2020 China Family Panel Studies. The findings indicate that consumption inequality has a significant negative impact on SWB. Specifically, for every unit increase in consumption inequality, the probability of individuals rating their SWB as “Happy” and “Very happy” decreases by 0.37% and 5.45% respectively. In addition, individuals’ confidence about their future serves as an intermediary in the connection between consumption inequality and SWB. The investigation of heterogeneity evidences that the adverse impact of consumption inequality on SWB is more pronounced in terms of subsistence and development expenditures. Consumption inequality affects SWB more seriously among lower-income and urban residents. Overall, this study holds important implications for addressing economic inequality to bolster individuals’ welfare.

## 1. Introduction

Human well-being has been attracting increasing attention from scholars, improving human well-being has become a prominent research topic in public policy and economics. Many studies have claimed that income is the most crucial factor that influences human well-being. However, since the “Easterlin paradox” was proposed [1], the relationship between well-being and income has been ambiguous. This paradox is also known as the “happiness–income puzzle”, which suggests that whereas there may be a positive correlation between individual well-being and income level in cross-sectional data [2], this relationship does not consistently increase with social and economic development or the national income level over time. Easterlin paradox implies that income alone cannot directly determine human well-being. Therefore, the number of studies that have explained the Easterlin paradox by considering other dimensions of economic inequality has been increasing.

Research on well-being primarily focuses on subjective well-being (SWB) [3], which is a person’s comprehensive judgment of their overall life satisfaction by using self-determined standards [4]. Many studies have explored the effect factors of SWB from the perspective of income inequality. However, a study by Aguiar and Bils (2015) investigated the trends of income inequality and the evolution of consumption inequality in the United States since 1980. They showed that these trends are not always consistent while they may be similar during certain periods, they diverge at other times [5]. Moreover, people derive more emotional satisfaction and value from the relative quantity and consumption. Thus, consumption is a better indicator of individual living standards and welfare [6, 7]. In developing countries, consumer expenditures are deemed a more comprehensive precise indicator of economic resources [8]. Although the income gap is a significant driver of consumption inequality, consumption inequality is also influenced by elements such as consumption preferences, tendencies, and liquidity constraints. Consumption can also be indicative of an individual’s actual economic strength, as it is more visible than income, which can be hidden. Therefore, it is necessary to assess the effect of consumption inequality on people’s welfare and SWB.

Sanfey and Teksoz (2007) demonstrated that income inequality is related to a higher degree of life fulfillment in transitioning countries, but in advanced and rich nations, it correlates with lower life satisfaction [9]. Moreover, a recent study indicated that in lower-income nations, people’s SWB is negatively affected by inequality, whereas in higher-income nations, it is significantly influenced by democratic quality and inflation [10]. Nevertheless, understanding SWB in China is challenging for it is necessary to consider factors such as urban-rural gaps and regional differences [11]. Thus, analyzing the relationship between consumption inequality and the SWB of Chinese residents can deepen the current understanding of the economy and society of China and other developing countries. Consumption inequality, as a fundamental factor affecting individual behavior and psychological perception, raises curiosity about its specific impact on SWB. Are there differences in the impact of various types of consumption expenditure? Which groups are most affected by consumption inequality in terms of SWB? Accordingly, this study addresses these questions to shed light on how consumption inequality influences individuals’ SWB.

This study enriches the existing literature with the following contributions. First, it focuses on consumption inequality rather than income or asset inequality, which enriches the study of factors influencing SWB. Second, it differs from prior studies that have focused on inequality at the regional level, this study explores the relationship between consumption inequality and SWB at the household level. Last, this study investigates the heterogeneity and the mechanisms by which consumption inequality relates to SWB, which is valuable for improving individuals’ SWB.

The remainder of this paper is organized as follows. Section 2 offers an overview of existing literature. Theoretical considerations and hypothesis formulation are the focus of Section 3. Section 4 details this study’s data sources, variables, methods and modeling strategies. The empirical analysis results are presented in Section 5. Last, Section 6 presents the conclusions and policy insights of this study.

## 2. Literature review

Since the Easterlin paradox was first proposed in 1974, the topic of happiness or well-being has received widespread scholarly interest and such research has developed rapidly in the economics. In psychology and other social sciences, well-being is categorized into “subjective well-being” and “objective well-being” and is frequently used as an indicator of psychosocial health [12–14]. SWB reflects people’s subjective judgment of their whole existence, whereas objective well-being focuses on indicators of poverty and income [15, 16]. Diener et al. (1985) characterized SWB as the comprehensive appraisal of one’s life satisfaction, judged by self-set measures, which are subjective, stable, and holistic [4].

In academic research, SWB is typically assessed in terms of happiness [17, 18]. It is usually measured through respondents’ answers, employing a type of self-assessment score, to respond to questions such as “What level of happiness do you feel?” [19, 20]. Some researchers have noted that the presence of confounding factors may affect the scores, and hence, the genuine intrinsic value of happiness scores cannot be determined using this approach [21, 22]. However, others have shown that these self-reported happiness questionnaires are currently the most effective method for gathering data on SWB, and the information contained in the data is more substantial than confounding factors, making these questionnaires a reliable and valid tool for use in empirical studies [23].

Studies on individual SWB have primarily focused on the factors that influence SWB. Most studies have indicated that poverty significantly and substantially affects citizens’ well-being [24], and that a rise in income is connected with increased happiness for persons living during the same period both in developed and developing nations [17, 25]. Recent studies indicated that even adolescents’ SWB is strongly linked to their family’s economic status and consumption expenditure [26]. Although marginal happiness decreases as income levels increase, the influence of income on happiness retains its significantly positive impact [27]. Nevertheless, prosperity in income does not invariably cause an escalation in happiness [28]. Numerous studies have attempted to explain the Easterlin paradox by considering relative income and wealth distribution imbalances. Most of these studies suggested that when income increases at different rates for different groups of people, those whose relative income decreases are inclined to harbor less happiness with life in the face of relative comparisons with peers, whereas those whose relative income increases do not necessarily see a significant increase in their satisfaction with life [29, 30]. Other studies drew the opposite conclusion and suggested that income inequality and the wealth gap increase individuals’ SWB [6, 31]. Moreover, several researchers concluded that economic disparity does not affect SWB [14, 32, 33]. In addition, scholars have analyzed other influencing factors of SWB, such as social capital inequality, unemployment, inflation, donation behavior, urban amenities, and ecological system [34–37].

A growing body of literature has indicated that income distribution alone may not adequately capture all aspects of social inequality and may be unsuitable as a welfare measure for all societies [38]. Consumption is considered a more accurate indicator of permanent income when liquidity limitations, precautionary savings, and other considerations are included [39]. When analyzing the factors that influence happiness, it should be noted that consumption and income should not be perceived as interchangeable factors [40]. Consumption inequality should be considered an essential dimension when studying social inequality [41]. Consumptive behavior might be a more precise indicator of an individual’s well-being since it can reflect one’s access to long-term resources, such as loans or public insurance [42].

Scholars have found that material consumption may contribute to a beneficial effect on SWB [43, 44]. The consumption of gifts instills a beneficial and statistically robust effect on SWB, mediated by the construct of social trust [45]. Furthermore, the relationship between consumption levels and happiness could follow an upside-down U pattern: consumption within a specific limit can enhance perceived happiness, but excessive consumption may reduce happiness [46]. However, some scholars have asserted that people are more concerned about relative consumption levels and that the factor influences life satisfaction is the person’s luxury consumption spending as compared to the average within the reference community, rather than their expenditure on essential goods or services [47]. Relative consumption has been found to be negatively correlated with participants’ assessments of their household well-being [48]. Lei et al. (2018) used data from China to study how inequalities affect the life satisfaction of Chinese residents. They indicated that in China, expenditure, rather than income, is a better measure, and expenditure inequality is negatively associated with life satisfaction [49].

In summary, numerous studies have focused on the factors that influence SWB, but primarily from the perspective of income, to reveal the effects of income inequality on individuals’ life satisfaction or happiness, using insights from relative deprivation and the psychology of social comparison. The latest research has shown that income is significant in meeting people’s physiological needs but is less crucial in satisfying their safety, belonging, esteem, and self-actualization needs [50]. Nevertheless, Maslow’s structure of need prioritization indicates that in addition to fulfilling physiological needs, humans seek fulfillment in terms of safety, belonging, esteem, and self-realization. Accordingly, directing all attention to the effects of wealth level on SWB might result in other essential determinants being overlooked. Consumer expenditure can satisfy people’s desire for social identity because it is more visible and open to comparison than income levels, which can be hidden. Consequently, consumer behavior may better satisfy people’s social, belonging, and esteem needs. In addition, an individual’s spending habits give more insight into their standard of living than their income alone, as it encompasses not only their income level but also their social security, borrowing ability, and other relevant information. Therefore, as a crucial perspective to analyze elements that affect SWB, more research on consumption inequality is needed and the relevant theoretical achievements need to be further enriched.

## 3. Theoretical analysis and research hypotheses

### 3.1 The effect of consumption inequality on SWB

Individual life satisfaction and SWB decrease owing to social comparisons and relative deprivation [51, 52]. People tend to feel depressed if they compare themselves to peers who are better off (i.e., upward comparison) [53]. The primary aspect influencing an individual’s utility is their consumption expenditure. According to the relative income hypothesis [54], people’s consumption expenditures are not only related to their absolute income level but also easily affected by the consumption level of others, that is, the demonstration effect of consumption. Therefore, the main factor that affects consumers’ satisfaction is their relative consumption expenditure, rather than just their absolute consumption. Individuals will adjust the utility they derive from their current consumption by comparing it with that of others. Severe consumption inequality will affect consumer comparison, and higher level of consumption will not always serve as inspiration if consumers cannot keep up with it, and more often, a feeling of relative deprivation arises when they feel inferior in this comparison of economic standing [55]. Thus, increases in consumption inequality magnify the relative deprivation individuals feel and diminish their SWB. This analysis culminates in the proposition of the following hypothesis:

Hypothesis 1: Consumption inequality has a negative effect on SWB.

### 3.2 The mediating role of confidence in the relationship of consumption inequality and SWB

By definition, SWB is people’s self-evaluation of their living conditions, self-efficacy and SWB are closely intertwined. Moreover, cognitive beliefs play a crucial role in connecting personality traits with SWB, and those with higher self-trust tend to experience higher levels of SWB [56]. Hopes and fears about the future are essential mechanisms for the link between inequality and SWB [57]. Clark et al. (2009) indicated that according to Hirschman’s tunnel effect [58], individuals experience higher job satisfaction when the income level of other workers in the same unit is higher, as this might indicate that their own future salary will also increase [59]. However, they emphasized that it is crucial to recognize that the critical determinant of state-signal equilibrium is the correlation between an individual’s prospective earnings and the present earnings of their comparative group. This correlation is likely to be weak within homogeneous peer groups or regions. For example, people do not feel more confident about their future earnings because their neighbors receive a raise. One study even indicated the rise in neighbors’ financial gains could have an adverse bearing on people’s sense of well-being [60]. This effect probably occurs because individuals lack the confidence that they can keep up with the increasing rate of their neighbors’ income. In line with this discussion, this study presents the second hypothesis:

Hypothesis 2: Consumption inequality has a negative effect on SWB because it can decrease people’s confidence in their future.

### 3.3 Different consumption inequalities have varying effects on SWB

Following Maslow’s structure of need prioritization, individuals’ consumption demand has different levels of progression and demand intensity. For example, based on the comparative theory of psychology, conspicuous spending increases life satisfaction more than basic expenses [47]. However, as expenditure on food, housing, and durable goods can meet individuals’ most essential and basic needs, it plays a beneficial and notable role in enhancing life satisfaction [61]. What’s more, unlike luxury consumption, household expenditure on necessities directly affects the health and cognitive ability of household members. Meanwhile, expenditures on development, including spending on schooling and training, shape an individual’s educational degree and the future career development of individuals and thus ultimately affect their confidence in their future. Therefore, compared with consumption inequality in other expenditures, consumption inequality in subsistence and development expenditures may significantly affect individuals’ SWB. Based on this analysis, this study proposes the third hypothesis:

Hypothesis 3: Consumption inequality in subsistence and development expenditures has a greater effect on SWB than consumption inequality in other expenditures.

### 3.4 The effect of consumption inequality on SWB changes among diverse groups

Different groups’ cognition of SWB may differ because of their varying endowments, characteristics, and behavioral choices. Relative income information can enhance the well-being of those with high income but decrease that of low-income groups [62]. Furthermore, the adverse effect of comparative income on the latter group’s happiness is greater than its positive effect on the former’s happiness [63]. For people living in poverty, the impact on their well-being is even more significant because the perception of injustice and lower trust associated with inequality reduces their happiness [64]. As a direct result of income inequality, consumption inequality decreases the relative consumption levels of low-income groups, which results in their experiencing more relative deprivation than high-income groups. The confidence of low-income groups about the future is always fragile and thus vulnerable to the impact of consumption inequality. Accordingly, this study introduces the fourth hypothesizes:

Hypothesis 4: Consumption inequality exerts a greater effect on the SWB of low-income groups than on high-income ones.

## 4. Data, variables, and methods

### 4.1 Data

In this study, the data was derived from the China Family Panel Studies (CFPS) [65]. It is a nationwide, two-yearly research project managed by the Institute of Social Sciences Survey at Peking University, initiated in 2010. The CFPS encompasses a sample that spans 25 Chinese provinces and municipalities. It includes personal information databases such as education level, age, social security payment, and well-being, as well as household information databases, including on household income, property, and various consumption expenditures. Given that data on SWB are unavailable in CFPS 2016, This study used data from CFPS 2014, 2018, and 2020, ensuring there were no missing reports of individual well-being.

The CFPS surveyed 13946, 15051, and 13015 households in 2014, 2018, and 2020 respectively. We cleaned the original data by removing missing household consumption and other essential variables. Annual household consumption and income per capita were winsorized by 1% to avoid extreme values that may potentially change the empirical results. This study involved households whose heads were aged between 16 and 75 years to ensure the reliability of these respondents’ answers. Given that this study calculated consumption inequality at the county level, the samples of households from counties with fewer than 10 households were deleted to avoid a reduction in data reliability. After cleaning and matching the data, this study obtained 24313 valid samples.

### 4.2 Variables selection and statistic descriptions

#### 4.2.1 SWB indicator

The CFPS questionnaire measures SWB by asking respondents, “How happy are you?” They quantify the happiness level using a scale where 0 is the least SWB and 10 is the most. To align with other studies, this paper reassigned the scores using the Richter Five-Point Scale method: [0,2][3,4][5,6][7,8][9,10] was reassigned to 1,2,3,4,5, which mean “Unhappy at all” “Unhappy” “So-so” “Happy” and “Very happy” respectively. Table 1 presents the distribution of reported happiness levels for the CFPS participants. In all, a majority of 64.98% of the surveyed individuals claimed that they were feeling happy or very happy with their lives, contrasting with the 7.67% who expressed not being happy or unhappy at all. This study also found that 68.25% of urban residents reported being happy or very happy, which exceeds the 61.97% reported in rural areas. Additionally, those with a higher level of income are usually happier than those in the low-income group, given that only 4.59% of the highest-income group but 11.99% of the lowest-income group self-reporting a lack of happiness.

**Table 1:** Distribution of Chinese residents’ SWB by residence and income.

Subjective well-being	Total	Urban	Rural	Lowest-income	Lower-middle income	Upper-middle income	Highest-income
Unhappy at all (%)	3.09	2.45	3.68	5.26	3.05	2.44	1.70
Unhappy (%)	4.58	4.00	5.11	6.73	4.66	4.10	2.89
So-so (%)	27.36	25.31	29.24	32.43	29.46	25.35	22.16
Happy (%)	35.29	39.02	31.86	27.48	32.85	36.98	43.88
Very happy (%)	29.69	29.23	30.11	28.10	29.97	31.12	29.38
*N*	24313	11644	12669	5702	6520	6311	5780

#### 4.2.2 Consumption inequality measures

Consumption inequality can be described using indices like the Gini coefficient and the Theil index from the macroscopic perspective. However, from the perspective of individual welfare, under the influence of the comparison effect or keeping up with the Joneses, most people tend to overlook general social inequality and are inclined to perform a narrow social comparison, that is, they are more concerned about relative deprivation [66]. Relative deprivation is the degree of inequality that an individual perceives when comparing themselves to others who are superior to them. The indicators of consumption inequality from macroscopic aspects are the Yitzhaki, Kakwani, and Podder indices. Compared with the Yitzhaki and Podder indices, the Kakwani index overcomes the defects of normalization and dimensionless [67]. Therefore, this paper employed the Kakwani index to describe household consumption inequality in China. The Kakwani index was calculated as follows:

$$Consumption\_inequality_{i}=\frac{1}{n\mu_{X}}(n_{c_{i}}^{+}\times \mu_{c_{i}}^{+}-n_{c_{i}}^{+}\times c_{i})=\frac{1}{\mu_{X}}\gamma_{c_{i}}^{+}(\mu_{c_{i}}^{+}-c_{i})$$ (1)

Where *Consumption*_*inequality*_*i*_ is the extent of consumption inequality in household *i* as quantified by the Kakwani Index; *μ*_*X*_ is the average consumption level of all households in the group *X*; $n_{c_{i}}^{+}$ is the amount of households in the group whose household consumption expenditure exceeds $c_{i};\mu_{c_{i}}^{+}$ is the average consumption expenditure of households whose expenditure exceeds $c_{i};\gamma_{c_{i}}^{+}$ is the percentage of households in group *X* whose consumption level exceeds *c*_*i*_. The Kakwani index has the following characteristics: it is a decreasing function of household consumption. That is, the lower the consumption level, the greater the consumption relative deprivation index, which indicates that the degree of deprivation is more serious. The Kakwani index ranges from 0 to 1.

Given that the county has been a constant administrative tier throughout Chinese historical periods [6], and there is a unique sense of affiliation with the county among Chinese citizens, which makes the measurement of inequality at the county level meaningful. Therefore, this study employs the Kakwani index of per capita household expenditure at the county level to measure consumption inequality. Table 2 reports that the mean consumption Kakwani index value is 0.405, while the income Kakwani index is 0.412, which indicates that household consumption inequality is slightly lower than income inequality. The result is consistent with those of current studies [68], which suggests that our data processing is reliable. The per capita annual consumption expenditure of a household is the aggregate household consumption outlay for the year, divided by its household population. The total consumption expenditure includes the sum of household equipment and daily essentials, clothing, shoes and hats, culture, education and entertainment, food, housing, medical care, transportation and communication, and other expenditures.

**Table 2:** Descriptive statistics and variable definitions.

variable	Definition	Mean	Std dev	Min	Max
*Subjective well—being*	Unhappy at all = 1, Unhappy = 2, So-so = 3, Happy = 4, Very happy = 5	3.839	1.003	1	5
*Consumption_inequality*	Kakwani index of annual per capita expenditure of household	0.405	0.231	0	0.959
*Income_inequality*	Kakwani index of annual per capita income of household	0.412	0.250	0	1
*Confidence*	with no confidence at all = 1, with no confidence = 2, So-so = 3, with confidence = 4, with full confidence = 5	4.091	0.975	1	5
*LogAverage_expense*	The logarithm of average expenditure of all household in a county	9.637	0.432	8.399	11.110
*Gender*	Male = 1, Female = 0	0.517	0.500	0	1
*Age*	Age of respondents	45.277	11.11	16	75
*Age* ^2^	Age squared	2173.48	986.51	256	5625
*Edu*	Illiterate/semi-literate = 1, Primary school = 2, Junior high school = 3, Senior high school /Vocational school = 4, Junior college = 5, University = 6, Master = 7, Doctor = 8	2.853	1.355	1	8
*Spouse*	Married / Cohabitation = 1,	0.872	0.334	0	1
	Cohabitation / Divorce / Widowed = 0				
*Health*	Very healthy = 1, healthy = 2, So-so = 3, Not healthy = 4, Not healthy at all = 5	3.005	1.205	1	5
*Insurance*	Having at least one kind of endowment insurance = 1, Having not any kind of endowment insurance = 0	0.716	0.451	0	1
*Hsize*	Number of household members	3.979	1.822	1	21
*Hukou*	Urban = 1, Rural = 0	0.479	0.500	0	1
*LogIncome*	The logarithm of household annual per capita net income	9.523	1.132	0	14.514
*LogAsset*	The logarithm of household total asset	12.304	2.946	0	25.329
*LogDebt*	The logarithm of household total debt	4.134	5.399	0	16.133

#### 4.2.3 Other explanatory variables

The intermediate variable in this study was people’s confidence in their future. The CFPS set the question for respondents, “How confident are you about your future?” utilizing a scale from 1 to 5, where 1 is the lowest and 5 is the highest. The responses reflect people’s expectations for the future. Table 2 reports that the average value of the sample’s confidence in their future is 4.091, which implies that a significant portion of the respondents hold a positive outlook for their future. Moreover, according to studies on the individual SWB, factors such as per capita household income, family size, household registration type, gender, age, education, marital status, physical health, and social security can influence SWB [17, 45]. Therefore, these variables were considered family and individual control variables, while the per capita household consumption expenditure in the county served as the county control variable. Table 2 provides the details of the definitions and statistical descriptions for these variables.

### 4.3 Correlations test

For a preliminary verification of the relationship between consumption inequality and SWB, this paper conducted a correlation test between consumption inequality, individuals’ confidence about their future, and SWB. Table 3 demonstrates that consumption inequality has a significant negative correlation with SWB. From the perspective of different consumption expenditure types, this paper found that subsistence expenditure inequality and development expenditure inequality also have a strong and significant negative correlation with SWB, while entertainment expenditure inequality has a relatively weak relationship with SWB. Additionally, this paper investigated the relationship between confidence and the core variable. The results suggest that there exists a strong positive correlation between confidence and SWB, while a significant negative correlation is evident between confidence and consumption inequality. This is consistent with the hypothesis we proposed above.

**Table 3:** Correlation test.

variable	*Subjective well-being*	*Consumption inequality*	*Subsistence expenditure inequality*	*Development expenditure inequality*	*Entertainment expenditure inequality*	*confidence*
*Subjective well-being*	1.000					
*Consumption inequality*	-0.057***	1.000				
*Subsistence expenditure inequality*	-0.076***	0.865***	1.000			
*Development expenditure inequality*	-0.054***	0.402***	0.219***	1.000		
*Entertainment expenditure inequality*	-0.011*	0.676***	0.400***	0.170***	1.000	
*confidence*	0.387***	-0.027***	-0.044***	-0.024***	0.014**	1.000

Note:

***P<0.01

**P<0.05

*P<0.1

### 4.4 Methods

The data used for empirical analysis in this study comes from CFPS, a survey that involves direct interactions with human participants. According to the official website, of CFPS, the full name of the ethics committee that approved the study is “Committee Member of Biomedical Ethics, Peking University.” The approval number is IRB00001052-14010, and the form of consent obtained is verbal.

The empirical analysis of SWB typically involves two methods. One approach is to take SWB as a ranked variable and utilize a ranking multiple-choice model, like the ordered probit model. The alternative is to take SWB as a numerical variable and employ the ordinary least squares (OLS) regression model. The OLS and ordered probit models formed nearly identical estimated results [63]. In addition, the coefficient interpretation of the OLS model is more intuitive [6]. Therefore, this paper employed two methods for testing: pooled OLS and ordered probit. The ordered probit model was employed in this study to analyze the marginal effect. Following the preceding methodology and theoretical analysis, this study sets SWB as the dependent variable, the key independent variable is consumption inequality. The mathematical model is presented in the following manner:

$$SWB_{it}=\beta_{0}+\beta_{1}\;Consumption\_inequality_{it}+\sum_{j=1}^{n}\gamma_{j}CV_{jit}+\mu_{i}+\lambda_{t}+\varepsilon_{it}$$ (2)

where subscript *i* represents the respondent; subscript *t* refers to time; *SWB*_*it*_ is the dependent variable, signifying the SWB of the respondent *i* at time *t*; *Consumption*_*inequality*_*it*_ refers to the consumption Kakwani index of household *i* at time *t*; *CV*_*jit*_ is a cluster of control variables; *μ*_*i*_ and *λ*_*t*_ serve as dummy variables for the fixed effects of years and counties, respectively; and *ε*_*it*_ is an unobserved white noise disturbance. To reduce the impact of heteroscedasticity, this paper utilized a cluster-robust standard analysis at the county level in the regression and took the logarithms for variables including the average consumption expenditure, per capita net income, assets, and household debt.

## 5. Result

### 5.1 Consumption inequality and SWB

Table 4 displays the estimated results on how consumption inequality is related to SWB using both pooled OLS and ordered probit models. we employed year and county fixed effects regression and adopted cluster analysis at the county level. The two methods are convenient for comparing and assessing robustness.

**Table 4:** The effects of consumption inequality on SWB.

Dependent variable	*Subjective well-being*			
Models	pooled OLS	ordered probit	pooled OLS	ordered probit
	(1)	(2)	(3)	(4)
*Consumption_inequality*	-0.2179***	-0.2190***	-0.1479***	-0.1691***
	(0.0316)	(0.0348)	(0.0346)	(0.0398)
*Gender*			-0.0642***	-0.0764***
			(0.0139)	(0.0158)
*Age*			-0.0500***	-0.0552***
			(0.0045)	(0.0051)
*Age* ^2^			0.0006***	0.0006***
			(0.0001)	(0.0001)
*Spouse*			0.4364***	0.4707***
			(0.0248)	(0.0262)
*Insurance*			0.0631***	0.0667***
			(0.0161)	(0.0185)
*Edu*			0.0205***	0.0142*
			(0.0067)	(0.0076)
*Health*			-0.1613***	-0.1893***
			(0.0058)	(0.0069)
*Hukou*			0.0017	0.0021
			(0.0197)	(0.0227)
*Hsize*			0.0322***	0.0353***
			(0.0046)	(0.0053)
*LogIncome*			0.0741***	0.0803***
			(0.0072)	(0.0079)
*LogAsset*			0.0089***	0.0093***
			(0.0022)	(0.0025)
*LogDebt*			-0.0100***	-0.0110***
			(0.0013)	(0.0015)
*LogAverage_expense*			0.1350**	0.1498**
			(0.0635)	(0.0733)
*Cons*	3.9893***		2.8298***	
	(0.0174)		(0.5913)	
*Time*.*FE*	YES	YES	YES	YES
*County*.*FE*	YES	YES	YES	YES
*N*	24313	24313	24313	24313
*R*^2^ */ PseudoR*^2^	0.0450	0.0171	0.1220	0.0472

Note: The values within parentheses are the robust standard errors, clustered at the county level.

***P<0.01

**P<0.05

*P<0.1

The dependent variable of Models (1) to (4) is SWB. In Models (1) and (2), this study used only the consumption Kakwani index as an independent variable and did not include control variables. In Models (3) and (4), this study included individual, family, and county control variables. The results for Models (1) to (4) indicate that the coefficient of consumption inequality is negative and statistically significant at the 1% level. These results indicate that consumption inequality significantly decreases individuals’ SWB. Moreover, Models (3) and (4) demonstrate that the adverse impact of consumption inequality on SWB exceeds the positive contributions of education, endowment insurance, family size, income, and assets on SWB. Table 5 displays the marginal effect of consumption inequality on SWB as estimated by the ordered probit model. The analysis reveals that for each unit rise in consumption inequality, the probability of an individual’s SWB being ranked “Not happy at all” increases by 1.07%, “Not happy” increases by 1.17%, “So-so” increases by 3.59%, “Happy” decreases by 0.37%, and “Very happy” decreases by 5.45%. The empirical evidence proves that consumption inequality negatively correlates with SWB, which is consistent with Hypothesis 1.

**Table 5:** The marginal effect of consumption inequality on SWB.

*Subjective well-being*	Unhappy at all	Unhappy	So-so	Happy	Very happy
Marginal	0.0107***	0.0117***	0.0359***	-0.0037***	-0.0545***
Effect	(0.0025)	(0.0028)	(0.0085)	(0.0009)	(0.0127)

Note: The values within parentheses are the robust standard errors, clustered at the county level.

***P<0.*a*01

**P<0.05

*P<0.1

In terms of the influence of other control variables on SWB. Table 4 indicates that having at least one kind of endowment insurance can significantly boost individuals’ SWB. The effect of household size, income, assets, education, and average consumption expenditure in the county on SWB is positive, while debt has a negative effect. The type of household registration (*Hukou*) does not affect SWB. Females tend to have greater SWB than males. The relation between an individual’s age and their SWB is characterized by a U-shaped curve, the elderly and the young have higher SWB, and the age where the curve inverts is about 42 years (0.05/(2×0.0006)≈42). Those having a spouse are happier than those who are single. The healthier the people, the happier are they. These findings regarding the influence of these individual and family characteristics on individual SWB almost match those of prior studies [35, 36]. This illustrates that this study’s results are stable and dependable.

### 5.2 Consumption inequality, confidence, and SWB

This study adopts a step-by-step method to examine the mediating role of confidence between consumption inequality and SWB. The results in Table 6 indicate that consumption inequality reduces SWB by damaging people’s confidence in their future. The intermediate effect constitutes 36.42% of the overall effect ((-0.1402×0.3842)/(-0.1479)≈0.3642), thus supporting Hypothesis 2. Consumption inequality has a significant effect on residents’ confidence about their future. This finding suggests that an increase in consumption inequality is linked to a reduction in people’s optimism about life and to lower SWB.

**Table 6:** The mediation of confidence.

Dependent variable	*Subjective well-being*	*Confidence*	*Subjective well-being*
Models	(1)	(2)	(3)
*Consumption_inequality*	-0.1479***	-0.1402***	-0.0991***
	(0.0346)	(0.0318)	(0.0339)
*Confidence*			0.3482***
			(0.0082)
*Controls*	Yes	Yes	Yes
*Time*.*FE*	Yes	Yes	Yes
*County*.*FE*	Yes	Yes	Yes
*N*	24313	24313	24313
*R* ^*2*^	0.1221	0.1179	0.2259

Note: The values within parentheses are the robust standard errors, clustered at the county level. The control variables’ coefficients are not displayed in this table.

***P<0.01

**P<0.05

*Pa<0.1

### 5.3 Endogenous analysis

In an empirical analysis, the baseline regression model may be affected by endogenous bias caused by the inverse causal effect and the omission of unobservable variables. To address these issues, this paper utilized the interaction terms of post station quantity during China’s Ming Dynasty at the provincial level, the annual national tertiary industry investment, and individuals’ party identity, as the IV and applied the 2SLS method. A reasonable IV needs to satisfy both externality and correlation criteria, and historical data are usually a good choice. For example, Duranton and Turner (2012) used historical highway maps and early railroad maps of the United States to predict the distribution of modern highways [69]. Individuals’ consumption behavior is closely related to the level of transport infrastructure [70]. During the Ming Dynasty, post stations were typically constructed in areas with favorable topographical and geological conditions. Post stations were a subsidiary facility of the national transportation system and were usually located along major traffic routes. As long-distance trade and commerce developed, commercial towns began to form around some of these stations. The development of modern transportation in China has been influenced by this historical factor, leading to a correlation between consumption inequality and China’s Ming Dynasty post stations, which meet the relevant conditions. In addition, as more than 400 years have passed since the construction of these post stations, it is difficult to directly affect the SWB of contemporary people, which thus satisfies the exclusion restriction. However, the data structure of the Ming Dynasty post stations is in the form of a cross-section, making it unsuitable for direct use in a quantitative panel data analysis. To address this issue, this study followed Nunn and Qian’s (2014) processing method [71], in which they introduced a variable that changes over time to construct a panel tool variable. In this paper, the annual national tertiary industry investment is multiplied by it to create an interaction term that serves as an instrumental variable for province-level consumption inequality in that year. Furthermore, as the province-level instrumental variables may introduce bias and this study’s research dimension is at the individual-level, we used the household head’s party membership status to multiply the number of Ming Dynasty post stations in each province. On the one hand, different political identities may possess various social resources and mobility constraints, which would affect the consumption structure and expenditure of households, satisfying the assumption of correlation with consumption inequality. On the other hand, political identity does not directly affect individual happiness [72], which satisfies the exogeneity hypothesis.

Table 7 shows the endogenous analysis of the 2SLS models. The first stage of the 2SLS analysis shows that the IV is significantly correlated with consumption inequality, and the value of *F-*statistic exceeds 10, suggesting that the IV possesses significant explanatory power. The *p-*value of the Kleibergen‒Paap rk LM statistic is 0.0087, resulting in the strong rejection of the underidentification hypothesis. The Kleibergen-Paap rk Wald *F-*statistic exceeds the critical threshold of 6.66 for 20% bias, and thus, the hypothesis of weak IV was rejected. The significance and sign of the consumption Kakwani index remain unchanged in the 2SLS estimation results, implying that the above conclusion remains firm even after accounting for endogeneity.

**Table 7:** The results of endogenous analysis.

Dependent variable	*Consumption_inequality*	*Subjective well-being*
Models	First of 2SLS	Second of 2SLS
*Consumption_inequality*		-7.0518**
		(3.2606)
*IV*	-0.0000**	
	(0.0000)	
*Controls*	YES	YES
*Time*.*FE*	YES	YES
*County*.*FE*	YES	YES
*N*	16,183	16,183
*F* statistic	2114.78	
Underidentification test	Kleibergen-Paap rk LM	
	*F* = 6.890, *p* = 0.0087	
Weak identification test	Kleibergen-Paap rk Wald *F* = 6.820	

Note: The values within parentheses are the robust standard errors, clustered at the county level.

***P<0.01

**P<0.05

*P<0.1

### 5.4 Robustness results

In this section, the robustness test was conducted by changing the measurement methods of core variables and adjusting the sample size. As presented in Table 8. first, we transformed the described form of SWB. In the original CFPS questionnaire, SWB was described as having a score ranging from 0 to 10. In Model (1), this study analyzed SWB empirically using the original measure form. In Model (2), SWB was transformed into a 0–1 variable, whereby we reassigned [0,5] to “0”, which indicates “Unhappy”, and [6,10] to “1”, which indicates “Happy”, and this study adopted the logit fixed effect model for regression. The results remain significant and consistent. Second, we employed other indices to express consumption inequality, and Models (3) and (4) used the Yitzhaki index and the Podder index, respectively, for regression. The Kakwani index was recalculated at the province level in Model (5) to verify the robustness of the geographical selection range. Last, the robustness of sample selection was verified by constructing balanced panel data in the Model (6). The robustness test confirmed that using the Kakwani index to measure consumption inequality is reliable for investigating its impact on SWB.

**Table 8:** Robustness test.

Dependent variable	*Subjective well-being*					
Models	Change the measure way of dependent variable		Change the measure way of independent variable			Change sample
	Measure SWB from 0–10	Measure SWB from 0–1	Yitzhaki/1000	Podder	Kakwani index at the province level	balanced panel data
	(1)	(2)	(3)	(4)	(5)	(6)
*Consumption_inequality*	-0.3341***	-0.4435***	-0.0061***	-0.0803***	-0.1519***	-0.1986***
	(0.0784)	(0.0911)	(0.0018)	(0.0201)	(0.0361)	(0.0537)
*Controls*	Yes	Yes	Yes	Yes	Yes	Yes
*Time*.*FE*	Yes	Yes	Yes	Yes	Yes	Yes
*County*.*FE*	Yes	Yes	Yes	Yes	Yes	Yes
*N*	24313	24313	24313	24313	24313	10824
*R*^2^ */ PseudoR*^2^	0.1248	0.0933	0.1218	0.1220	0.1220	0.1356

Note: The values within parentheses are the robust standard errors, clustered at the county level. The control variables’ coefficients are not displayed in this table.

***P<0.01

**P<0.05

*P<0.1

### 5.5 Heterogeneity analysis

Considering that diverse consumer products, each serving distinct purposes, correspond to various levels of Maslow’s hierarchy of needs, this paper divided household expenditure into three categories: subsistence, development, and entertainment expenditure. Subsistence expenditure includes four categories: clothing, food, housing, and transportation. Development expenditure refers to cultural or educational consumption. Entertainment expenditure contains three categories: household equipment or daily necessities, medical care, and other consumption.

Table 9 shows the effect of inequality in various types of consumption on SWB. The empirical results indicate that inequality in subsistence and development consumption expenditures significantly reduces SWB. Moreover, the effect of subsistence consumption inequality on SWB is greater than that of development consumption inequality, while the coefficient of enjoyment consumption inequality is negative but not statistically significant. These results support Hypothesis 3 and show that consumption inequality in subsistence and development expenditures has a more pronounced effect on SWB. This difference is probably due to the fact that subsistence consumption like food, housing, dress, and transportation, and development consumption expenditure like culture and schooling, meet individuals’ immediate utility more effectively and are helpful for family members’ future development. Thus, inequality in subsistence and development expenditures is more likely than inequality in entertainment expenditure to cause individuals to lose confidence in the future, which ultimately reduces their SWB. Furthermore, this finding allows us to discern which needs people are more concerned about and indicates that consumption contains more information than income, which is a pivotal reason for this study focusing on consumption inequality instead of income inequality.

**Table 9:** The impact of inequality of different types of consumption on SWB.

Dependent variable	*Subjective well-being*					
Models	Subsistence expenditure		Development expenditure		Entertainment expenditure	
	pooled OLS	ordered probit	pooled OLS	ordered probit	pooled OLS	ordered probit
	(1)		(2)		(3)	
*Consumption*_*x*_ *_inequality*	-0.1792***	-0.1998***	-0.0705***	-0.0782***	-0.0277	-0.0382
	(0.0333)	(0.0381)	(0.0213)	(0.0247)	(0.0260)	(0.0302)
*Controls*	Yes	Yes	Yes	Yes	Yes	Yes
*Time*.*FE*	Yes	Yes	Yes	Yes	Yes	Yes
*County*.*FE*	Yes	Yes	Yes	Yes	Yes	Yes
*N*	24313	24313	24313	24313	24313	24313
*R*^2^ */ PseudoR*^2^	0.1224	0.0474	0.1217	0.0471	0.1213	0.0469

Note: The values within parentheses are the robust standard errors, clustered at the county level. The control variables’ coefficients are not displayed in this table.

***P<0.01

**P<0.05

*P<0.1

Table 10 presents the results regarding how consumption inequality affects SWB across various groups. Per capita household income is categorized as a ranked discrete variable numbered 1 to 4 in the CFPS, with 1 corresponding to the lowest income quartile while 4 to the highest. The lowest-income and lower-middle-income households were integrated into the low-income group, and remainder were integrated into the high-income group. Models (1) and (2) indicate that consumption inequality significantly diminishes SWB across all groups, but the effect is greater in the low-income group. Thus, Hypothesis 4 was supported. It is likely that the low-income group’s confidence in the future is more susceptible to external influences.

**Table 10:** The effects of consumption inequality on SWB in different groups.

Dependent variable	*Subjective well-being*							
Models	low-income		high-income		Urban		Rural	
	Pooled OLS	Ordered Probit	Pooled OLS	Ordered Probit	Pooled OLS	Ordered Probit	Pooled OLS	Ordered Probit
	(1)		(2)		(3)		(4)	
*Consumption_inequality*	0.1632***	0.1733***	0.1349***	0.1715***	0.2013***	0.2522***	0.1039**	0.1079***
	(0.0500)	(0.0534)	(0.0447)	(0.0564)	(0.0483)	(0.0578)	(0.0467)	(0.0513)
*Controls*	Yes	Yes	Yes	Yes	Yes	Yes	Yes	Yes
*Time*.*FE*	Yes	Yes	Yes	Yes	Yes	Yes	Yes	Yes
*County*.*FE*	Yes	Yes	Yes	Yes	Yes	Yes	Yes	Yes
*N*	12222	12222	12091	12091	11644	11644	12669	12669
*R*^2^ */ PseudoR*^2^	0.1229	0.0475	0.1256	0.0507	0.1307	0.0523	0.1292	0.0499

Note: The values within parentheses are the robust standard errors, clustered at the county level. The control variables’ coefficients are not displayed in this table.

***P<0.01

**P<0.05

*P<0.1

Furthermore, the economy of China has a unique urban-rural structure, and residents’ welfare is tied to their household registration (*Hukou*) type. Therefore, it is required to discuss the heterogeneity in the effect of consumption inequality on SWB under different household registration types. Models (3) and (4) show that consumption inequality significantly reduces SWB in both urban and rural areas. Moreover, it has a more severe effect on the SWB of residents who live in urban areas, which may be related to the fact that urban residents generally experience higher pressure and a faster pace of life than rural residents, they are more uncertain about their future. Hence, consumption inequality has a larger effect on their SWB.

## 6. Conclusion

This study used CFPS data to explore the effects of consumption inequality on SWB and conducted endogeneity analyses and robustness tests to confirm the findings. This study focused on the differences in these effects across different consumption expenditure types and investigated potential influencing mechanisms. The findings imply a significant negative relationship between consumption inequality and SWB in China. In addition, individuals’ confidence in their future serves as a mediator between consumption inequality and SWB, proving that individuals’ cognitive beliefs are vital in enhancing their happiness. Furthermore, this study examined how this effect changes across different categories of consumption and groups. We found that for the various consumption expenditure types, different expenditure inequalities have different effects on SWB. Specifically, subsistence and development expenditure inequalities have significant effects on people’s SWB, while entertainment expenditure inequality does not have a significant impact. Moreover, among the various income and household registration groups, the effect of consumption inequality on SWB is more severe in low-income and urban groups.

This study contributes the following findings and suggestions. First, consumption inequality, as an essential manifestation of social and economic development, directly affects people’s SWB. Increased consumption inequality undermines individuals’ confidence in their future, thus lowering their SWB. It is recommended that people form rational consumption concepts, maintain a positive outlook on life, refrain from engaging in unwarranted comparisons and overspending, and strive to enhance their SWB.

Second, confidence acts as a mediator between consumption inequality and SWB. When people feel uncertain and worried about the future, it can lower their overall assessment of their current lives, leading to a decrease in SWB. From the person dimension, individuals can improve their confidence and SWB by drawing up plans for their future work and life and taking steps to achieve these plans. From the government dimension, it is important to enhance income redistribution systems and mechanisms. For example, broadening social security coverage in China can help alleviate residents’ worries and pressures about the future and enhance their happiness.

Third, the effect of consumption inequality on reducing SWB is significant in subsistence and development expenditure but is not significant in entertainment expenditure. The adverse impact of consumption inequality on SWB is more severe among low-income and urban residents than for high-income and rural residents. Thus, policymakers should focus on promoting equal opportunities to ensure that people’s basic survival and development needs are equally met. Further, it is necessary to prioritize the needs of low-income groups, as their SWB is more adversely affected by consumption inequality.

Nevertheless, this study has some limitations. First, although self-report questionnaires have been widely used to measure SWB, happiness is multidimensional and complex. To enhance the accuracy and dependability of the results, it would be beneficial for subsequent research to incorporate multidimensional psychological measurement tools for a more detailed comprehensive assessment of SWB. Second, this paper discussed only one possible mechanisms by which consumption inequality affects SWB because of data and technical limitations. Consequently, future studies can discuss in depth other mechanisms through which consumption inequality affects SWB.

## Supporting information

S1 Appendix Selected questions from the CFPS questionnaire. (DOC)

S1 Dataset (DTA)
