# Supplementary material for: The effect of consumption inequality on subjective well-being: Evidence from China
Source: PLoS One. 2024 Nov 19;19(11):e0310193. doi: 10.1371/journal.pone.0310193 (PMC11575769; doi:10.1371/journal.pone.0310193)
Supplement: S1 Appendix — (DOC) [file pone.0310193.s001.doc]

**Table 11** **Selected questions from the CFPS questionnaire**

| Code | Question | Answer |
| --- | --- | --- |
| QM2014 | How happy are you? | ［0...10］(0 is the lowest, 10 is the highest) |
| FP3 | On average, what was the total amount spent by your household on food, snacks, drinks, tobacco, alcohol, etc.? | Exact expenses |
| FP501 | Over the last year,what was the total amount spent by your household on clothes, shoes and hats? | Exact expenses |
| FP502 | Over the last year, what was the total amount spent by your household on cultural entertainment, including books, magazines, going to the movies and theater? | Exact expenses |
| FP503 | Over the last year, what was the total amount spent by your household on travel? | Exact expenses |
| FP504 | Over the last year, what was the total amount spent by your household on central heating? | Exact expenses |
| FP505 | Over the last year, what was the total amount spent by your household on property (including parking and sanitation)? | Exact expenses |
| FP506 | Over the last year, what was the total amount spent by your household on repairs and renovations house? | Exact expenses |
| FP507 | Over the last year, what was the total amount spent by your household on the car? | Exact expenses |
| FP508 | Over the last year, what was the total amount spent by your household on purchase and maintain other means of transportation (such as bicycles, electric bicycles), communication tools (such as mobile phones) and accessories? | Exact expenses |
| FP509 | Over the last year, what was the total amount spent by your household on buy or maintain furniture, appliances and other consumer durables such as cars, computers, appliances, jewelry, antiques, high-end Musical Instruments, etc.? | Exact expenses |
| FP510 | Over the last year, what was the total amount spent by your household on education? | Exact expenses |
| FP511 | Over the last year, what was the total amount spent by your household on medical care? | Exact expenses |
| FP512 | Over the last year, what was the total amount spent by your household on health care? | Exact expenses |
| FP513 | Over the last year, what was the total amount spent by your household on haircuts and beauty treatments (including cosmetics, treatments, massages, etc.)? | Exact expenses |
| FP514 | Over the last year, what was the total amount spent by your household on commercial insurance, such as medical insurance, auto insurance, home and property insurance, commercial life insurance, etc.? | Exact expenses |
| RESP1 | Which member of your family is most familiar with your family’s income and expenses over the past 12 months? | Exact expenses |
| QP201 | How do you describe yourself health？ | (1)Very health; (2)Rather health; (3)Moderately healthy; (4)Not very health; (5)Unhealth |
| edu | The highest education of the respondent: | (1)Illiterate /semi-literate; (2)Primary school; (3)Junior high school; (4)Senior high school /Vocational school ; (5)Junior college; (6)University; (7)Master; (8)Doctor; (9) Never went to school |
| BC6 | The type of household registration is: | (1)Agriculture(Rural); (2)Non-Agriculture (Urban ); (3)Not registered |
| QI301 | What kinds of endowment insurance of the respondent insured for? | 1. Receive retirement funds from the organs or institutions; (2)Basic pension insurance; (3) Company-sponsored retirement insurance; (4) Commercial endowment insurance; (5)Rural pension insurance; (6)New rural social pension insurance; (7)Urban residents pension insurance; (8) Others; (9) None of the above |
| Fmlcount | Family size of respondent | Specific amount |
| FINC | Over the last year, What is the overall income for your household, considering business revenue, salary income, rental income, government allowances, and financial support from non-family members? | Exact expenses |
| BC4 | The marital status of the respondents is: | (1)Never married; (2)Have a spouse (in marriage); (3) Cohabitation; (4)Divorced; (5)Widowed |
| QN12016 | How confident are you about your future? | ［1...5］(1 is the lowest, 5 is the highest) |

Source: CFPS2014, 2018 and 2020
